# Stress, Depression, and Purpose-Specific Use of Dietary Supplements and Over-the-Counter Preparations: A Cross-Sectional Study of 120,000 Polish Adults

**DOI:** 10.3390/nu18213615

**Published:** 2026-10-30

**Authors:** Akanksha Urbanowicz, Anna Zimny-Zając, Mateusz Dróżdż, Grzegorz Mazur, Siddarth Agrawal

**Affiliations:** 1 Department of Emergency Medical Service, Wroclaw Medical University, 51-616 Wroclaw, Poland; akanksha.urbanowicz@gmail.com (A.U.);; 2 Medonet, Ringier Axel Springer Poland, Domaniewska St. 49, 02-672 Warsaw, Poland; 3 Faculty of Health Science, Institute of Public Health, Jagiellonian University Medical College, 31-066 Krakow, Poland; 4 Labplus R&D, Wyspa Słodowa 7, 50-266 Wrocław, Poland; 5 Faculty of Medicine, Andrzej Frycz Modrzewski Krakow University, 30-705 Kraków, Poland

**Keywords:** dietary supplements, over-the-counter preparations, stress, psychological well-being, depression, sleep, self-medication, nutritional epidemiology

## Abstract

Background/Objectives: Dietary supplements and over-the-counter (OTC) preparations are widely used to self-manage stress, low mood, and disturbed sleep. Population research has treated such use as one undifferentiated behaviour. We examined whether stress and affective burden co-occur with supplement and OTC use in a purpose-specific way and how they relate to self-medication and primary-care contact. Methods: We analysed cross-sectional data from the National Test of Poles’ Health (non-probability, self-selected web survey; wave 5, 2024; *N* = 120,000), with trend analyses across five cross-sections (2020–2024). Survey-weighted logistic regression related four indicators of psychological burden to purpose-specific use of mood-, sleep-, and memory-targeted products, with a symptom-by-purpose matrix, age- and sex-stratified replication, and E-values. Results: Mood-product use rose along a gradient of psychological burden, from frequent stress (adjusted odds ratios [aOR] 1.80, 95% confidence intervals [CI] 1.72–1.88) through work-related exhaustion (2.17, 2.08–2.26) and poor self-rated mental health (2.44, 2.34–2.55) to a lifetime depression diagnosis (2.53, 2.41–2.65). A lifetime depression diagnosis also co-occurred with sleep (1.92, 1.83–2.01) and memory-product use (1.77, 1.69–1.85), while multivitamin and hair/skin use stayed flat (aOR ≈ 1.0). In this subgroup (*n* = 21,793), targeted use co-occurred with OTC self-medication (mood 1.70, 1.59–1.83; sleep 1.61, 1.50–1.73) without reduced primary-care contact. Associations were observed in both broad age strata (≤54 vs. >54 years) and both sexes (E-values 4.3–9.5). Purpose-specific use rose across the five cross-sections. Conclusions: Supplement use tracks stress and affective burden in a purpose-specific way and, among respondents reporting a lifetime physician diagnosis of depression, accompanies OTC self-medication. This pattern marks a group whose self-management concentrates on preparations with limited trial support. The scope of these findings is set by two features: the depression measure is lifetime, not an assessment of current symptoms, and the questionnaire recorded the purpose of use, not the product identity, ingredients or dose.

## 1. Introduction

Depressive disorders are among the most prevalent and disabling health conditions worldwide, affecting an estimated 280 million people and ranking as a leading contributor to years lived with disability [1]. Although effective treatments exist, the majority of affected individuals remain untreated; rates of minimally adequate care range from approximately 25% in high-income countries to just a few percent in low- and lower-middle-income settings [2]. This treatment gap is increasingly filled by self-management, whereby individuals address symptoms such as low mood, sleep disturbances, and stress either independently of or alongside formal healthcare services. Nutrition sits at its centre. Psychological stress reshapes eating behaviour, increasing the intake of energy-dense foods and reducing the intake of healthier ones [3], and nutritional strategies—from whole-diet change to single-nutrient supplementation—are among the first actions people take when psychological well-being deteriorates. Characterising which nutritional products people reach for, and for which stated reason, is therefore a question for nutrition and public health as much as for psychiatry.

Dietary supplements and OTC preparations are among the primary modalities of this self-management, reflecting a broader trend where interest in nutrition and nutraceuticals as contributors to mental health has consolidated into a distinct field [4]. The evidence base, however, is highly variable and, for most products, modest. A meta-review of meta-analyses of randomised controlled trials found robust support for eicosapentaenoic acid–rich omega-3 supplementation as an adjunctive treatment for depression, no support for any nutrient as a standalone or first-line treatment, and little compelling evidence for the most widely consumed vitamins and minerals [5]. Even for more extensively studied products, pooled effects are small and heterogeneous: omega-3 supplementation yields a modest reduction in depressive symptoms [6], and exogenous melatonin shortens sleep-onset latency by an average of several minutes [7]. Consequently, a scoping review of OTC preparations for depression concluded that research has concentrated on a narrow range of agents, contrasting with the persistent consumer drive toward self-management [8]. A substantial disconnect therefore exists between consumer utilisation and empirical support, with direct implications for how supplement use should be interpreted in patients presenting with affective symptoms.

Despite these implications, population-based research on supplement use has largely treated it either as a single, undifferentiated behaviour or through its sociodemographic determinants. Recent nationally representative Polish surveys characterised supplement use by age, sex, education, and dietary pattern [9,10]. These studies explore who uses supplements and what they take. However, they offer limited insight into the psychological dimensions of this behaviour. Whether—and to what extent—stress and affective burden co-occur with the use of products targeted at mood, sleep, or cognition, and whether such associations are purpose-specific or reflect a generalised propensity toward supplement consumption, remains unexamined at the population level. In this study, ‘affective burden’ operationalises the self-reported psychological and stress-related constructs evaluated here—namely, poor self-rated mental health, frequent perceived stress, and work-related exhaustion, alongside symptoms such as insomnia and memory complaints—examined in tandem with a lifetime depression diagnosis.

Among the limited studies that have incorporated mental health metrics, the evidence remains highly heterogeneous. Analysis of data from the US National Health and Nutrition Examination Survey (NHANES) indicated that adults with active depressive symptoms had lower odds of dietary supplement use, whereas those taking prescription antidepressants exhibited higher odds [11]. By contrast, a Saudi survey reported markedly higher supplement use among respondents with a prior psychiatric diagnosis and with insomnia [12]. One candidate contributor to this divergence is how the mental-health construct is operationalised: a current symptom screen and a reported clinical diagnosis capture different points along the path from distress to care and need not agree in direction; in the European Health Interview Survey in Spain, a self-report item of this kind had a sensitivity of 52.9% and a positive predictive value of 40.4% against a concurrent symptom screen [13]. This motivates large samples that index a lifetime depression diagnosis explicitly, while being clear about the limits of such a measure.

The purpose a respondent states for taking a supplement or OTC product is itself a nutrition-behaviour variable: it marks where nutritional self-management is directed when psychological well-being deteriorates, towards single-nutrient and botanical preparations [5,6,7] and away from whole-diet change. Resolving use at this level locates, at population scale, the point at which dietary counselling and medication-safety review would have to be aimed. We address these gaps using the National Test of Poles’ Health (NTZP), to our knowledge, one of the largest European survey programmes of its kind, comprising approximately 120,000 adults in its most recent wave and repeated cross-sections spanning 2020–2024—an order of magnitude larger than the Epidemiology of Mental Disorders and Access to Mental Health Care study (EZOP), Poland’s reference psychiatric-epidemiological survey of roughly 10,000 adults [14]. The same programme has been used to describe a behaviourally distinct consumption profile, in which energy-drink use clustered with smoking, poorer diet, and burnout [15]; supplements taken for symptomatic relief and stimulant beverages are different forms of consumption, and the affective dimension of supplement use is a separate question, addressed here. We aimed to (i) describe the prevalence and frequency structure of supplement and OTC use; (ii) estimate the associations of frequent stress, work-related exhaustion, and broader affective burden with purpose-specific use of mood-, sleep-, and memory-targeted products; (iii) assess the specificity of these associations across a full symptom-by-purpose matrix; (iv) characterise, among respondents reporting a lifetime physician diagnosis of depression, the co-occurrence of targeted use with OTC self-medication and current primary-care contact; (v) assess whether the pattern is observed in both broad age strata (≤54 vs. >54 years) and in both sexes, and (vi) describe temporal trends from 2020 to 2024. We hypothesised that psychological burden is associated with supplement and OTC use in a purpose-specific way, graded across the severity of that burden and concentrated on mood-, sleep-, and memory-targeted products.

## 2. Materials and Methods

### 2.1. Study Design and Participants

**Figure 1:**
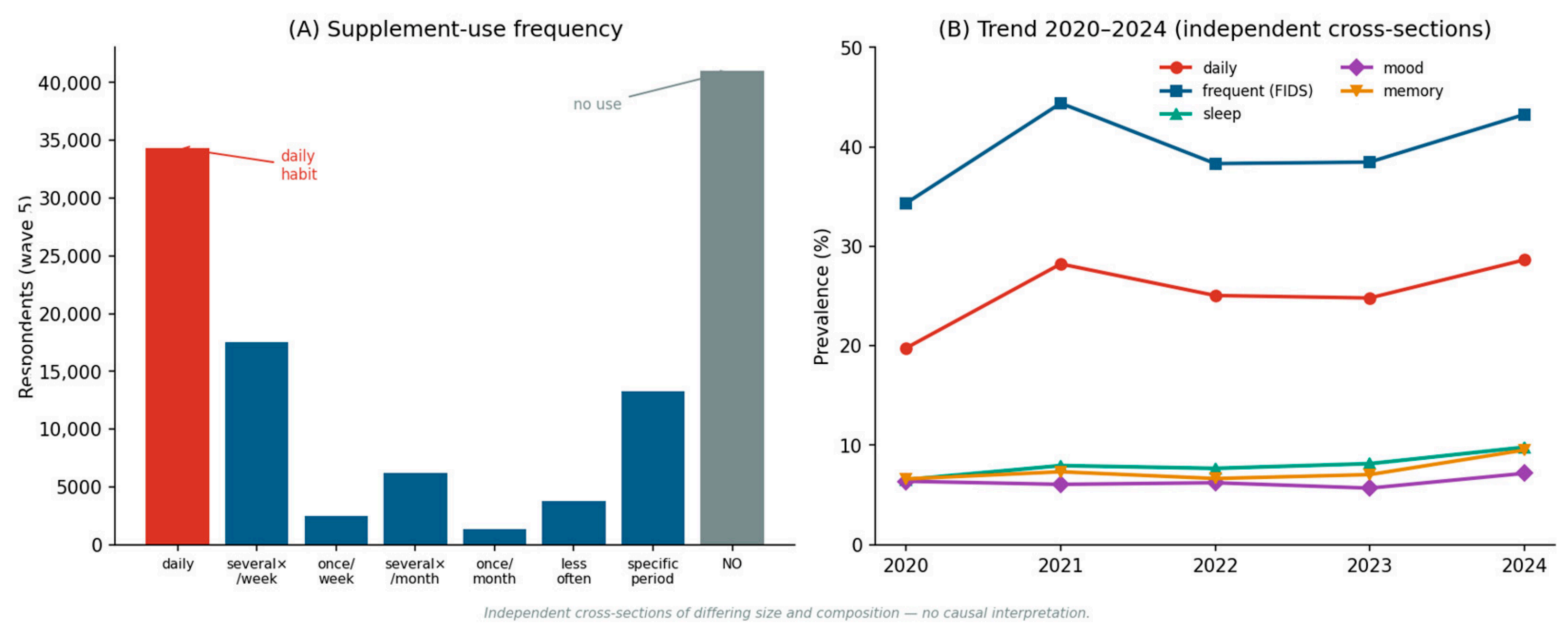
Frequency structure (**A**) and 2020–2024 trend (**B**) of supplement use. (**A**) Distribution of self-reported supplement-use frequency (wave 5, 2024; *N* = 120,000), showing a bimodal pattern with modes at daily use and no use and a trough across intermediate categories, supporting dichotomisation. Bar colour distinguishes the two modes (red, daily use; grey, no use) from the intermediate categories (blue) and encodes no additional variable. (**B**) Prevalence of daily use, frequent use (FIDS), and mood-, sleep-, and memory-targeted use across five independent cross-sections, 2020–2024 (descriptive and unweighted; independent samples of differing size and composition).

We analysed cross-sectional data from the National Test of Poles’ Health (Narodowy Test Zdrowia Polaków, NTZP), a self-administered, web-based computer-assisted web interviewing (CAWI) survey of Polish adults conducted annually from 2020 to 2024 (waves 1–5) using a non-probability, self-selected sampling approach, with participants recruited through the Medonet health portal (Ringier Axel Springer Polska). All adjusted cross-sectional models were estimated on the most recent wave (wave 5, 2024; *N* = 120,000). The 2024 wave comprised the full NTZP file of 120,000 complete responses and was analysed in full; no records were excluded, filtered, or subsampled at the analysis stage. Although the NTZP programme comprises five annual cross-sections (2020–2024), these represent independent samples that cannot be linked at the respondent level and differ substantially in size and composition; they therefore were not pooled for the adjusted models. Waves 1–4 span the COVID-19 pandemic, which independently elevated both supplement use and the prevalence of depressive and anxiety symptoms [16,17]; restricting estimation to the single post-pandemic 2024 wave avoids embedding this period confounding. Given the size of the 2024 wave (statistical power ≈ 1, with already-narrow confidence intervals), additional observations from earlier waves would not materially improve precision while reintroducing compositional and period heterogeneity. We therefore use wave 5 as a single, internally consistent, contemporary cross-section, and the 2020–2024 waves descriptively (Figure 1B).

Trend analyses additionally used the 2020–2024 records, treated as independent repeated cross-sections of differing size and composition (wave N ranging from approximately 376,000 in 2020 to 120,000 in 2024). The NTZP is a voluntary, anonymous online survey of adult internet users. Before starting the questionnaire, participants were briefed on the objectives of the survey, the nature of the data to be collected, and the data-handling procedures, and all provided informed electronic consent. For the 2024 data collection, the Bioethics Committee of the Military Chamber of Physicians in Warsaw reviewed work on this survey programme and determined that formal ethical approval was not required (decision no. KB 65/2024). The present work is based exclusively on the fully anonymised archival 2024 dataset, with no recontact of respondents and no new data collection; this secondary analysis was approved by the Bioethics Committee of the Lower Silesian Medical Chamber in Wrocław (resolution no. 24/BNBO/2025 of 10 September 2025). Sex was respondent-reported (woman/man). Reporting follows the Strengthening the Reporting of Observational Studies in Epidemiology (STROBE) guidance for cross-sectional studies [18].

The NTZP programme supports diverse health-behaviour analyses. Three studies draw on the same programme: energy-drink consumption and its lifestyle correlates [15]; self-perceived health literacy in the 2024 wave [19]; and the sociodemographic, lifestyle and work-related correlates of mental-health outcomes across all five waves [20]. The present analysis addresses a different research question, with non-overlapping exposures, outcomes, and conclusions. In particular, the present study resolves supplement and OTC use by each product’s stated purpose and shows the affective association to be purpose-specific and discriminant.

### 2.2. Measures

#### 2.2.1. Supplement and OTC Use

Respondents first reported the frequency of dietary supplement and OTC preparation use over the past year on eight ordered categories (item: “Did you take dietary supplements or over-the-counter preparations in the past year?”; 1 = daily, 2 = several times a week, 3 = once a week, 4 = several times a month, 5 = once a month, 6 = less than monthly, 7 = only for a defined period, 8 = no use), reverse-coded so that lower values denote more frequent use. Those reporting any use then indicated, from a multiple-response list, the kind and purpose of products used (item: “What kind of, and for what purpose, did you take dietary supplements or OTC preparations in the past year?”). We derived purpose-specific binary indicators from this list: mood-targeted use from “to improve mood and well-being”, sleep-targeted from “preparations that aid falling asleep”, and memory-targeted from “to improve memory and concentration”, alongside multivitamin, hair/skin, eyesight, digestion, weight-loss, and potency categories. “Purpose” thus reflects the respondent’s self-reported reason for use—a behavioural construct—not a product’s ingredient profile. The questionnaire covered dietary supplements and over-the-counter preparations under a single stem and recorded no product name, brand, ingredient, dose, duration of use, regulatory category, or concomitant prescription medication. References to supplement use without further qualification denote this combined category. We derived three a priori frequency contrasts: any use in the past year, daily use, and frequent use (frequent intake of dietary supplements, FIDS, at least several times a week, i.e., the two most frequent categories). The frequency distribution was markedly bimodal, with modes at “daily” and “no use” and a trough across intermediate categories, supporting dichotomisation and a “supplementation-as-habit” framing.

#### 2.2.2. Mental-Health and Symptom Exposures

A lifetime depression diagnosis, self-rated mental health, stress frequency and symptom frequency were all self-reported single items from the survey programme, not adapted from validated multi-item instruments. A lifetime physician diagnosis of depression was taken from a yes/no disease checklist (item: “Have you ever been diagnosed with any of the following diseases/conditions?”), in which depression was one row (1 = yes, 2 = no). The item is lifetime (“ever diagnosed”) and records the fact of a diagnosis without its date, the current activity of the condition, treatment status, or its position in time relative to the reported product use. Self-rated mental health came from a single comparative item (“Overall, how do you rate your mental health compared with others your age?”; poor = the lowest two of five categories). Frequent stress was defined from “How often do you experience stressful situations?” (at least several times a week). Frequent symptoms—insomnia, memory problems, and work-related exhaustion—were defined as occurring “at least several times a month” on a symptom-frequency item (“Which health complaints do you suffer from, and how often?”).

#### 2.2.3. Self-Management and Care-Contact Indicators

These indicators came from a separate part of the questionnaire, under a different stem, from a multiple-response coping item (“How do you most often cope when health complaints or illness arise?”): OTC self-medication from “I use over-the-counter medicines bought on my own”, and management on a physician’s advice from “I follow a doctor’s advice”. The current primary-care contact was a general-practitioner visit within the previous 6 months (“When were you last at a primary-care physician as a patient?”). Among respondents reporting a lifetime diagnosis, contact “ever” is near-universal by definition, so current contact is the discriminating indicator.

#### 2.2.4. Covariates

All models adjusted for a pre-specified covariate set covering four domains. Demographic characteristics were age (≤54 vs. older, and continuous in sensitivity analyses) and sex. Socioeconomic position was captured by higher education and urban residence, the latter defined as a town of at least 100,000 inhabitants. Health status was entered as overweight or obesity (body-mass index ≥ 25) and multimorbidity (two or more chronic conditions). Lifestyle entered as current smoking, regular alcohol use (at least two to three drinks per week), weekly fast-food consumption, and weekly physical activity (at least one hour per week).

#### 2.2.5. Data Completeness and Validation

Coding was validated against pre-specified target prevalences before modelling; all check items reproduced to within 0.1 percentage points. Incomplete questionnaires and responses with implausibly short completion times were excluded at source during data collection. The analytic file therefore comprised complete questionnaires with no imputation [18]. Purpose-specific and outcome indicators were derived from multiple-response and single items and contained no missing values on analytic variables; for the multiple-response purpose, coping, and channel items, a non-selected option was coded 0, so the absence of missingness on these derived indicators is a property of the coding convention (“not selected = 0”)—a “0” on a purpose indicator therefore combines genuine non-use with non-endorsement. To ensure complete respondent anonymity, no identifying information (IP addresses, cookies) was collected at any stage, so abandoned or incomplete sessions were not recorded at source; a completion or attrition rate therefore cannot be computed for this design. Complete-case analysis retained the full *N* = 120,000 in every cross-sectional model.

### 2.3. Statistical Analysis

Associations were first screened with χ^2^, Mann–Whitney, and Kruskal–Wallis tests, then estimated with multivariable logistic regression. Survey-weighted models were the primary analysis. Population weights were normalised to a mean of 1 (sum of weights = N)—constructed by rim weighting (iterative proportional fitting) to the marginal distribution of adult Polish internet users (sex, age, educational attainment, and size of place of residence; Mediapanel benchmarks), with weights ranging from 0.37 to 6.0, as documented for this dataset elsewhere [20]—and a robust (sandwich, HC0) variance estimator, so confidence intervals are robust to heteroscedasticity; unweighted models are reported as sensitivity analyses (Supplementary Table S2). Weighting aligns the sample with the marginal distribution of adult Polish internet users and is used to assess robustness to sample composition instead of claiming representativeness; agreement between the weighted and unweighted estimates indicates that the associations are not artefacts of sample composition. For the core purpose-specific models, each of the three affective-relevant outcomes (mood-, sleep-, and memory-targeted use) was regressed on each mental-health or symptom exposure entered in a separate model with the covariate set above; exposures were not mutually adjusted. Frequent stress, work-related exhaustion, insomnia, poor self-rated mental health and a lifetime depression diagnosis are closely related constructs, and one may lie on the causal path to another; entering them in a single model would control for intermediate variables.

A descriptive model for frequent use (FIDS) was fitted for context with its discrimination (area under the curve) and calibration (Hosmer–Lemeshow) reported in the Supplementary Materials. Symptom-by-purpose specificity was summarised as a matrix of adjusted aOR (one model per symptom–purpose pair; cells with an exposed count < 50 excluded) and visualised as a heatmap, with the insomnia–sleep pairing as a pre-specified validity anchor. A self-management analysis within respondents reporting a lifetime physician diagnosis of depression regressed OTC self-medication and the care-contact indicators on mood- and on sleep-targeted use. Temporal patterns (2020–2024) were summarised as prevalence by wave and reported descriptively, without causal interpretation.

Sensitivity analyses comprised Benjamini–Hochberg control of the false-discovery rate across the core test pool; the minimum cell-count threshold; sex-stratified models for sex-sensitive products; age- (≤54 vs. >54) and sex-by-age replication (both unweighted); E-values for the principal associations, computed on each reported odds ratio with the rare-outcome formula appropriate to our low-prevalence supplement outcomes (and the risk-ratio approximation for the common FIDS outcome) following VanderWeele and Ding [21]; and continuous-age specifications. Age- and sex-stratified analyses and the trend were estimated unweighted. Given the large sample (power ≈ 1), interpretation emphasised effect size against a pre-declared threshold of |OR| ≥ 1.2, not statistical significance. All statistical analyses were performed in Python v3.12.3 (Python Software Foundation, Wilmington, DE, USA). Logistic regression models and Benjamini–Hochberg false-discovery rate correction were implemented with the statsmodels package v0.14.6 [22]. A pre-specified exploratory latent-class analysis of the purpose indicators, performed with the StepMix package v3.0.0, was unstable (low entropy) and is not reported.

### 2.4. Causal-Language Statement

Because all data are cross-sectional, findings are reported in associational terms only (co-occurrence, higher or lower odds). For each principal association, three readings remain indistinguishable in these data: (a) symptoms prompting supplement use; (b) supplement users more readily noticing or reporting symptoms; and (c) being in care with a diagnosis prompting added self-medication.

## 3. Results

### 3.1. Sample Characteristics

The wave-5 sample was predominantly female (62.7%) and middle-aged to older (mean age 57.7 ± 14.4 years; median 60; range 18–99; 39.0% aged ≤ 54) (Table 1). Affective and symptom burden was common; weighted estimates (the primary basis for inference) exceeded crude sample proportions for several affective indicators, reflecting calibration away from the older, female-skewed composition (weighted first, unweighted in parentheses): a lifetime depression diagnosis 20.0% (sample 18.2%), poor self-rated mental health 44.5% (37.0%), frequent stress 53.9% (46.0%), frequent insomnia 24.8% (27.2%), frequent work exhaustion 33.0% (26.1%), and frequent memory problems 12.2%. Any supplement use in the past year was reported by 65.4% (sample 65.8%), daily use 26.9% (28.6%), and frequent use 42.3% (43.2%); weighted purpose-specific use was 9.6% (mood), 9.2% (sleep), and 9.6% (memory). For managing illness, 29.3% reported OTC self-medication (sample 22.8%) and 42.0% managed on a physician’s advice; 60.4% had a primary-care visit within the previous 6 months.

### 3.2. Distribution of Use and Unadjusted Associations

The frequency of supplement use was sharply bimodal, with modes at daily use (*n* = 34,347; 28.6%) and no use (*n* = 41,001; 34.2%) and a trough across intermediate categories; the full eight-category distribution is given in Supplementary Table S1, with the largest intermediate category (“several times a week”) accounting for 17,536 respondents (14.6%) (Figure 1A). This supports dichotomisation and a “supplementation-as-habit” framing. Unadjusted associations previewed both an affective signal and on-purpose specificity: mood-product use co-occurred with a lifetime depression diagnosis (crude OR 3.02; 14.9% vs. 5.5%), sleep-product use with insomnia (4.98; 21.8% vs. 5.3%), and memory-product use with memory problems (2.41; 18.0% vs. 8.3%). Users of mood products reported worse self-rated mental health than non-users (median 3.0 vs. 2.0; Mann–Whitney *p* < 0.001; Kruskal–Wallis across frequency categories H = 990.4, *p* < 0.001).

### 3.3. Affective Burden and Purpose-Specific Supplement Use

In survey-weighted adjusted models (Table 2), a lifetime depression diagnosis co-occurred with targeted supplement use across product categories. Mood-product use showed the strongest, graded affective pattern: it co-occurred most strongly with a lifetime depression diagnosis (aOR 2.53, 95% CI 2.41–2.65) and poor self-rated mental health (2.44, 2.34–2.55) and progressively with work exhaustion (2.17, 2.08–2.26) and frequent stress (1.80, 1.72–1.88). Critically, the association was not confined to mood products: a lifetime diagnosis also co-occurred with sleep-product use (1.92, 1.83–2.01) and memory-product use (1.77, 1.69–1.85). Poor self-rated mental health followed the same cross-category pattern (sleep 2.04, 1.96–2.13; memory 1.57, 1.51–1.63). Unweighted estimates were similar (Supplementary Table S2; e.g., mood–depression 2.73, 2.60–2.88). Each estimate in Table 2 is the association of a single exposure with purpose-specific use at the level of the covariate set, with the mental-health exposures left unadjusted for one another.

### 3.4. Symptom-by-Purpose Specificity

**Figure 2:**
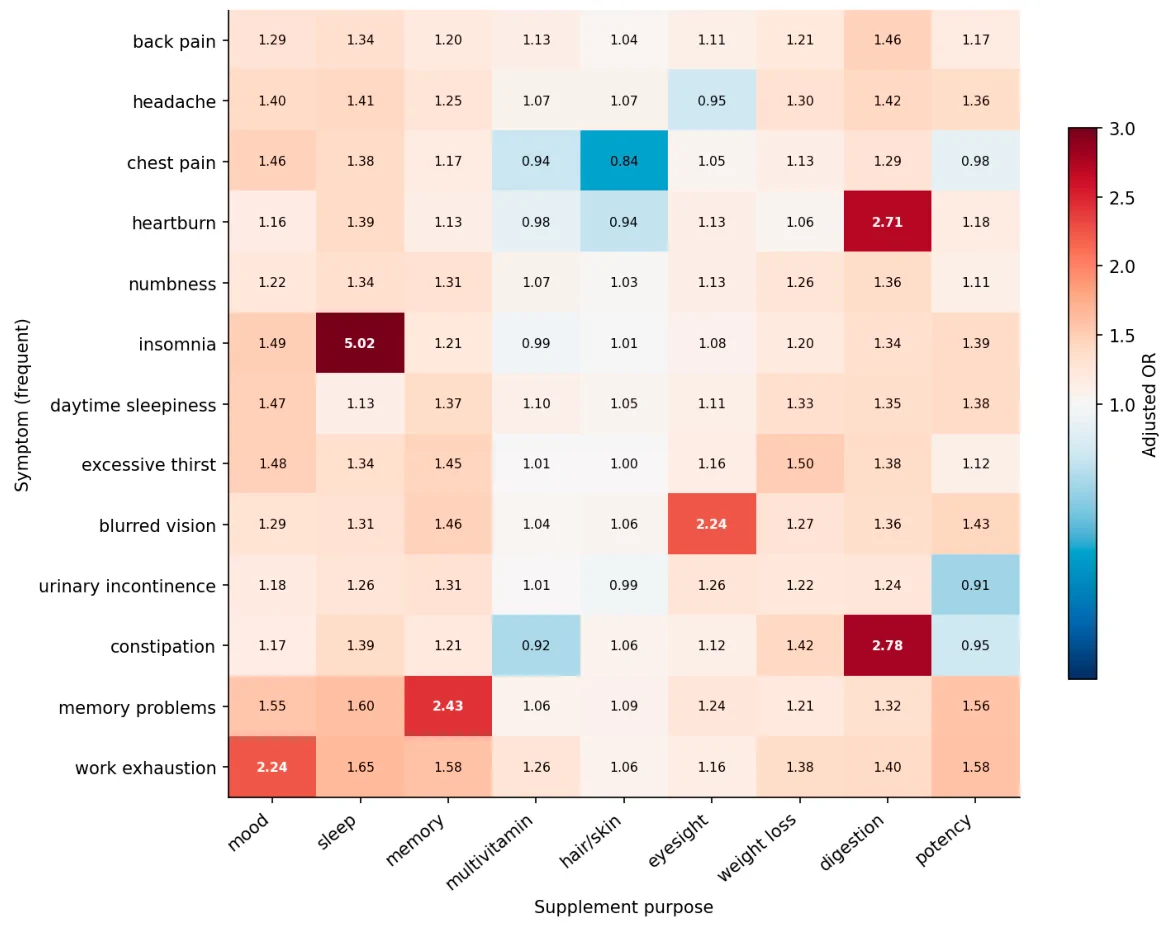
Symptom-by-purpose specificity matrix (adjusted odds ratios; wave 5; *N* = 120,000; unweighted models). Each cell is from a separate multivariable logistic model (purpose-specific use regressed on a frequent symptom, full covariate set); cells with an exposed count <50 are omitted. On-purpose pairs lie on the diagonal (convergent validity); the insomnia–sleep cell is a pre-specified validity anchor. Flat multivitamin and hair/skin columns indicate non-specific products (discriminant validity).

The full symptom-by-purpose matrix (Figure 2) showed the expected pattern of convergent and discriminant validity. On-purpose pairs were strongest along the diagonal, with somatic pairs as clear convergent anchors (heartburn and constipation with digestion products; blurred vision with eyesight products; memory problems with memory products, weighted aOR 2.31, 95% CI 2.20–2.43, Table 2). The insomnia–sleep pairing, pre-specified as a validity anchor, was the single strongest cell (weighted aOR 4.49, 4.31–4.69; 5.02 in the unweighted matrix of Figure 2); because this co-occurrence is expected, we treat it as confirmation that the matrix recovers on-purpose specificity rather than as a substantive affective finding. Discriminant validity was evident in the multivitamin and hair/skin columns, which were essentially flat across symptoms (aOR ≈ 1.0).

### 3.5. Frequent Supplement Use

Frequent use (FIDS) was associated with a broadly health-orientated profile—female sex, higher education, physical activity, and younger age—together with a lifetime depression diagnosis (weighted aOR 1.16, 1.12–1.20), multimorbidity, and symptoms, and inversely with current smoking (full model, discrimination, and calibration in Supplementary Table S3). Because this model is explanatory rather than predictive, its discrimination (area under the curve, AUC, 0.64) is not the relevant criterion; the positive depression direction is taken up in the Discussion.

### 3.6. Self-Management Among Respondents Reporting a Lifetime Physician Diagnosis of Depression

Among the 21,793 respondents reporting a lifetime depression diagnosis, mood- and sleep-product use co-occurred with OTC self-medication (weighted aOR 1.70, 1.59–1.83, and 1.61, 1.50–1.73). Neither product was associated with current primary-care contact (mood 0.96, 0.90–1.03; sleep 1.09, 1.01–1.18) or with management on a physician’s advice (both ≈ 1.0), providing no evidence of care substitution at the level of current contact. A combined pattern of affective-supplement use, OTC self-medication, and no current primary-care contact was about twice as common among respondents reporting a lifetime diagnosis (4.45%) as without (2.38%); within the diagnosis stratum, this sub-pattern was more common among supplement users (16.5%) than non-users (11.5%). A supplement-use × depression interaction for OTC self-medication was null (mood 1.00, 0.92–1.09), indicating a general pattern rather than a depression-specific one.

### 3.7. Robustness Across Age Strata and by Sex

The core associations with stress and affective burden were reproduced in both age strata (≤54 and >54 years) and in both sexes (Supplementary Table S4a,b; unweighted). The mood–depression association was present below 54 (aOR 2.46, 95% CI 2.29–2.63) and stronger above 54 (3.13, 2.90–3.39) and present in all four sex-by-age cells (range 2.31–3.30). The sleep–depression association held across strata (range 1.66–2.21), and the insomnia validity anchor was uniformly strong (range 4.54–5.27). The affective signal is therefore not an artefact of the older, predominantly female sample.

### 3.8. Temporal Trends, 2020–2024

Across the five independent cross-sections, daily use and frequent use rose over the period, as did the prevalence of purpose-specific products (sleep ≈ 6.5% to 9.8%; memory ≈ 6.6% to 9.5%; mood 6.4% to 7.2%, Figure 1B), while any use was essentially unchanged. Because the waves are independent cross-sections of differing composition, these patterns are descriptive only.

### 3.9. Sensitivity Analyses

Findings were stable across pre-specified checks (Supplementary Table S5). Of 135 tests in the core pool, 118 were significant at a Benjamini–Hochberg false-discovery rate of q < 0.05, of which 84 also met |OR| ≥ 1.2. E-values for the principal associations, computed on each reported odds ratio (rare-outcome formula for the supplement outcomes), were large—4.91 (CI bound 4.63) for mood–depression, 9.52 (9.11) for sleep–insomnia, 4.30 (4.07) for memory–memory problems, and 1.40 (1.35) for FIDS–depression (weighted: 4.49, 8.46, 4.04, and 1.36)—indicating that substantial unmeasured confounding would be required to explain away the strong associations; the E-value addresses unmeasured confounding only, not reverse causation. Continuous-age specifications differed negligibly (Δ aOR ≤ 0.12). One small, atypical cell (potency products with depression among women) was flagged and excluded from interpretation. All analytic variables were complete, so every model used the full sample.

## 4. Discussion

In this cross-sectional analysis of approximately 120,000 Polish adults, the use of dietary supplements and OTC preparations co-occurred with psychological burden in a strictly purpose-specific manner. This association followed a gradient across the severity of that burden: for mood-targeted products, frequent stress (weighted aOR 1.80), work-related exhaustion (2.17), poor self-rated mental health (2.44), and a lifetime depression diagnosis (2.53). These purpose-specific associations were markedly stronger than the baseline association between depression and frequent supplement use overall (FIDS; weighted aOR 1.16, 95% CI 1.12–1.20), which indicates that the signal reflects targeted utility, not a generalised propensity toward supplement consumption. The on-purpose associations along the matrix diagonal—anchored by the strong co-occurrence of sleep products with insomnia (weighted aOR 4.49)—are partly expected given that respondents self-reported their reasons for use. We therefore treat this diagonal as a convergent-validity anchor rather than a novel empirical finding.

Three features are less expected and constitute the contribution. First, an affective diagnosis co-occurred with use across product categories, extending beyond mood products: a lifetime depression diagnosis was associated with sleep-product use (aOR 1.92) and memory-product use (1.77) as well. Second, the gradient set out above shows that mood-product use tracks the intensity of psychological burden. Third, the associations were discriminant: the non-specific multivitamin and hair/skin columns were flat across symptoms (aOR ≈ 1.0), which argues against a general propensity to consume supplements. This pattern is also far from deterministic—only about 15% of respondents with a lifetime depression diagnosis reported mood-product use.

The co-occurrence of a lifetime depression diagnosis with sleep-product use aligns with the well-established, bidirectional relationship between insomnia and depressive disorders. Prospectively, insomnia roughly doubles the risk of developing subsequent depression [23,24] and serves as a robust predictor of incident depression, anxiety, and psychosis across cohorts [25]. Because sleep disturbance functions as both a core feature and a clinical prodrome of depressive illness, sleep aids are the most frequently consumed mental health supplements [12]. Notably, this widespread utilisation persists despite empirical evidence indicating that their average hypnotic efficacy is modest [5].

Our consistently positive direction—a lifetime depression diagnosis associated with higher odds of targeted and of frequent use—aligns with a Saudi survey in which a prior psychiatric diagnosis was associated with markedly higher supplement use [12], but runs opposite to NHANES, where current depressive symptoms were associated with lower odds [11]. These data cannot identify which factor drives the difference, and we do not attempt to adjudicate between the candidates. The two studies differ on several axes at once: setting and sampling (a probability-based US examination survey versus a Polish non-probability web panel) and measurement, since we indexed a single self-reported lifetime physician diagnosis, whereas NHANES used a validated current-symptom scale. A lifetime diagnosis and a current symptom screen capture different points on the path from distress to care and need not agree in direction [13]. One observation bears on this directly: our nearest symptom-like proxy, poor self-rated mental health, ran in the same positive direction as the diagnosis (aOR 2.44) and did not mirror the negative symptom association seen in NHANES. Measurement, setting and sampling therefore remain joint candidate contributors to between-study heterogeneity.

Among respondents reporting a lifetime depression diagnosis, mood- and sleep-product use co-occurred with OTC self-medication (weighted aOR 1.70 and 1.61), and a combined pattern of affective-supplement use, OTC self-medication, and no current primary-care contact was about twice as common as among those without a diagnosis. Purpose-specific use and OTC self-medication were elicited separately, under different stems and different reference frames: the first records what was taken over the past year and for what stated purpose, and the second what the respondent does when a health complaint arises. The co-occurrence is therefore not an artefact of measuring affect twice, although the behaviours themselves may overlap in the products involved. This combination describes a coherent self-directed management pattern, consistent with the well-documented drive to self-manage depressive symptoms where access to therapy is limited [8] and among older adults managing depression, anxiety, and insomnia with OTC preparations [26].

The self-management was nonetheless not one of disengagement from care: current primary-care contact was unrelated to supplement use in this stratum, and the supplement-self-medication association was not amplified by depression (interaction null), which indicates a general consumer pattern and not a depression-specific substitution of self-care for medical care. A recent Polish survey of 798 adults reached a convergent conclusion from a different angle [27]. Users of mood- and stress-relief supplements and OTC medications were no less likely than never-users to endorse general-practitioner consultation. Past users were more likely to endorse psychotherapy, although current users reported lower global trust in physicians (adjusted mean difference −0.40) and were less likely to raise product-related doubts with their doctor (relative risk ratio 0.52) [27]. Our estimates extend that picture from stated preferences in a sample of 798 respondents to observed care contact in approximately 120,000. The reduced willingness to disclose, documented in that study, set against the undiminished primary-care contact documented here, means that clinicians are seeing these patients and are unlikely to hear about the products unless they ask. The co-occurrence is equally compatible with a reverse reading in which a diagnosis and ongoing care prompt added self-medication.

An alternative, non-exclusive reading concerns who uses supplements. Frequent supplement users showed a health-orientated profile—more often women, better educated, physically active, and younger—matching the health-conscious supplement user described in earlier work and in recent Polish samples [9,10,28]. Such individuals may also monitor and report bodily and mood symptoms more readily, which would inflate the observed co-occurrence between symptoms and targeted use independently of any care-seeking. This surveillance-and-reporting reading is indistinguishable from a self-management reading in cross-sectional, self-reported data, and it is consistent with the positive symptom proxy noted above.

Daily and frequent supplement use and the prevalence of mood-, sleep-, and memory-targeted products rose across the five independent cross-sections. This accords with documented growth in supplement use during the COVID-19 period in Poland and globally [16], against a backdrop of a substantial pandemic-era rise in depressive and anxiety disorders, with the largest increases among women and younger adults [17]. These patterns are descriptive only, with no causal or pandemic-specific attribution.

Two strands of external evidence [5,6,7,27,29,30,31,32,33,34,35,36,37,38] make a patient’s use of mood- or sleep-targeted products worth eliciting during a complete clinical history. First, the products people most often use have, at best, modest and heterogeneous efficacy: among nutrient supplements, eicosapentaenoic acid (EPA)-predominant omega-3 is the single agent with robust adjunctive support in depression, with no nutrient supported as first-line or stand-alone treatment [5], and the pooled effects of omega-3 [6], vitamin D [29], probiotics [30,31], and melatonin [7] are small and condition-dependent, whereas whole-diet improvement has stronger support [32,33]. Second, supplement use carries safety and interaction implications that are often not disclosed to clinicians: herbal products can interact with serotonergic and other medications and contribute to hepatotoxicity [34,35,36], while a large minority of users do not report supplement use to their physicians [27,37]. A market perspective sharpens the point: preparations sold in Poland for mood improvement and stress relief carry producer and merchant claims that run ahead of the evidence supporting their ingredients [38]. The consequence for nutrition practice follows directly: purpose-specific use identifies a group whose self-management concentrates on preparations with the weakest trial support, while the dietary strategies with the strongest support sit outside the behaviour measured here. We deliberately refrain from recommending supplement use as a marker or screen for depression: at a population use prevalence near 10% and a depression prevalence near 20%, and without operating characteristics, such a use would not be justified.

Age entered the models as two strata (≤54 vs. >54 years), and the affective associations were reproduced on both sides of that threshold (mood-product use with a lifetime depression diagnosis: aOR 2.46 below 54 and 3.13 above). What this design does not resolve is the internal structure of the younger stratum, which spans early adulthood through the early fifties. The distinction matters because young adults carry a distinct and partly persistent burden from the pandemic period. Among Slovak adults aged 18–30, most reported health symptoms persisting after COVID-19, and physical inactivity compounded the deterioration in memory [39]. The pandemic footprint in this age group extended beyond mental health. Indicators of fat-free mass, skeletal muscle mass, total body water, and basal metabolic rate were lower in young men assessed during the pandemic than before it [40]; forearm bone quality was likewise lower during the pandemic, with bone mineral content lower in young men and vitamin D status an independent predictor of bone quality in young women [41]. Lifestyle factors also structure mood at this age: among 982 Slovak university students aged 18–30, sleep difficulties were the strongest correlate of both mental well-being and depressive symptoms, with physical activity, internet use, and smoking contributing further [42]. Two of these findings map directly onto the behaviour measured here—sleep difficulty as the dominant lifestyle correlate of mood, and vitamin D status as a nutritional correlate of a somatic outcome—since sleep- and nutrient-targeted products are precisely the categories at issue. Whether the purpose-specific pattern documented here takes the same form in early adulthood is an open question, and one that calls for age-resolved sampling.

The two product categories combined in the exposure item are neither pharmacologically nor legally equivalent. The boundary between them is porous in the market itself. Within the European Union the same ingredient may be marketed as a food supplement in one member state and as a medicinal product in another: melatonin is permitted in dietary supplements up to 2 mg in France, up to 1 mg in Poland, and not at all in the Czech Republic, and most ingredients questioned in the Polish notification procedure are substances licensed simultaneously under food and pharmaceutical law [43]. Consumers do not resolve what regulators leave unresolved. Among Polish adults, 40.3% classify dietary supplements as OTC medicines and fewer than 5% classify them correctly as food [44], and the confusion reaches the professions that advise them: 43% of Polish doctors, nurses and pharmacists describe dietary supplements as medicines or medical devices, and close to half believe the marketing requirements are the same as for medicinal products [45]. Knowledge is similarly uneven among Polish internet users, the population from which this sample is drawn [46]. Stated purpose is therefore the level at which this behaviour is organised for the consumer, and the level at which the present estimates operate. The point bears most on the sleep-targeted category, where supplement and medicine formulations of the same agents sit side by side on the same shelf, and on the self-management analysis, where the two indicators came from separate items whose underlying products may coincide.

Strengths of this study include the scale and granularity of the data, analysed at the level of each product’s stated purpose [9,10]. The convergent and discriminant validity checks, replication of the core associations across age strata and in both sexes, the survey-weighted primary analysis, and E-values quantifying robustness to unmeasured confounding [21] support the internal consistency of the affective signal. The large sample is a source of precision and not of representativeness: the estimates describe associations within this self-selected, internet-using sample.

Several considerations temper the “adjusted” interpretation. The lifestyle covariates were coarse (binary or trinary), leaving room for residual confounding, particularly by overall dietary pattern, on which supplement users tend to differ [28]. The corrected E-values bound this concern for the strong associations: an unmeasured confounder would need to be associated with both exposure and outcome by an odds ratio of roughly 4.5–9.5 (and about 1.4 for frequent use), beyond the measured covariates, to fully explain them. This bound applies to any factor outside the fitted models, measured or unmeasured.

The mental-health exposures entered separate models by design [47]. Each estimate is therefore specific to its own exposure, and the set should not be read as a decomposition of psychological burden into independent components [48]. Importantly, the E-value addresses unmeasured confounding only and does not address reverse causation.

### Limitations

Several limitations qualify these findings.

First and most importantly, the design is cross-sectional—direction cannot be established; for every principal association, three readings remain indistinguishable—symptoms prompting use, users more readily noticing or reporting symptoms, and being in care with a diagnosis prompting added self-medication—and reverse causation cannot be excluded.

Second, all exposures, symptoms, diagnoses, and use indicators were self-reported, raising the possibility of recall and common-method bias, including the symptom-monitoring reading above. Because the exposure (purpose-specific supplement and OTC use) and the outcomes (depression, symptoms, and self-medication) were drawn from the same self-report instrument, shared-method variance could inflate the associations [49]; the purpose-specificity of the pattern—sharp on-purpose peaks alongside flat non-specific columns and divergent magnitudes across exposures—argues against a uniform method artefact, although it cannot exclude one. The items have not undergone formal psychometric validation against reference instruments, and internal-consistency reliability is not estimable for a single-item measure; a validation study in a subsample is the natural next step for this programme.

Third, depression was captured by a single self-reported lifetime physician diagnosis (“ever diagnosed”). The item establishes that a diagnosis was made, but not when, whether the condition is currently active, whether treatment is ongoing, or how the diagnosis sits in time against the year of reported product use. Because depression is not necessarily lifelong, this category combines respondents in remission with respondents whose condition is currently active. That heterogeneity is non-differential with respect to the outcome and would be expected to attenuate the associations reported here, not to generate them. The estimates therefore describe how purpose-specific use co-occurs with a history of diagnosis, which is also where they part company with current-symptom studies such as NHANES.

Fourth, the questionnaire captured the purpose category of supplement use (multiple-response) and covered dietary supplements and OTC preparations under a single stem, without product names, brands, active ingredients, doses, duration of use, regulatory category, or concomitant prescription medication. This fixes the level at which the findings operate: they describe purpose-defined consumer categories, and inference about particular agents or supplement-drug interactions belongs to instruments that record composition. The same boundary applies to the self-management analysis, where purpose-specific use and over-the-counter self-medication came from separate items whose underlying products may coincide.

Fifth, the sample was a non-probability, self-selected web panel recruited through the Medonet health portal (CAWI), and the large N should not be mistaken for population representativeness. The sample is therefore likely enriched for health-engaged respondents, a pattern documented when volunteers are recruited for supplement research [50]; as health engagement may relate both to supplement and OTC use and to the likelihood of having received and reported a lifetime depression diagnosis, conditioning on participation could induce selection (collider) bias and distort the observed associations, not merely limit their generalisability. Only completed questionnaires were recorded at source, so any selection occurring during questionnaire completion cannot be assessed.

Sixth, the sample was skewed older (mean age 57.7 ± 14.4 years, median 60; 39% aged ≤ 54), and age entered the models dichotomously (≤54 vs. >54). The stratified estimates establish that the associations hold on both sides of this threshold, including in the working-age stratum and in both sexes, but do not resolve a finer age gradient: the ≤54 stratum spans early adulthood through the early fifties, age ranges that differ in mental health, lifestyle, healthcare utilisation, supplement use, and exposure to the persistent consequences of the pandemic period [39,40,41,42]. Separate conclusions regarding young adults, or more detailed age-related differences, fall outside what these data address. The programme enrols adults only.

Seventh, the covariate set was pre-specified and coarse, and characteristics outside it may confound the estimates. Diet quality is one such characteristic, since habitual intake and micronutrient data were not collected. Psychological burden accompanies a shift towards energy-dense foods [3], and lower diet quality accompanies lower engagement with supplementation [9,10,28]; a confounder with this structure would work against the positive associations reported here. The E-values quantify how strongly such a characteristic would need to be associated with both exposure and outcome to account for the principal associations.

Finally, the study is confined to a single country, and a pre-specified exploratory latent-class analysis was unstable and not relied upon; the conclusions rest on the purpose-specific models, which are independent of it.

## 5. Conclusions

In a large population-scale European sample, the use of mood- and sleep-targeted dietary supplements and OTC preparations co-occurred with stress and affective burden in a purpose-specific and graded manner. Additionally, the gradient was present from frequent stress and work-related exhaustion up to a lifetime depression diagnosis. Furthermore, among respondents reporting a lifetime physician diagnosis of depression, targeted supplement use co-occurred with OTC self-medication, without evidence of reduced concurrent primary care contact, which describes nutritional self-management alongside formal care. The depression measure is recorded over the whole lifetime, and this stratum therefore comprises people with a history of diagnosis; current symptom status lies outside what the survey measured.

The co-occurrences described above are compatible with a self-management response to psychological distress; they do not establish directionality. The survey identifies a population in which mood- and sleep-targeted use is common and concentrated on preparations that trials support less strongly than whole-diet approaches. The case for eliciting such use during a clinical history follows from the external evidence on efficacy, safety and disclosure reviewed above; the present estimates do not support supplement use as a screening marker for depression. Because the questionnaire recorded purpose of use and not product identity, ingredients or dose, these findings describe behavioural associations, and questions about particular nutrients, or about supplement-drug interactions, require instruments that record composition. Elucidating whether population-level supplement use precedes, follows, or accompanies affective burden remains a critical task for future longitudinal studies and linked-prescribing data.

## Figures and Tables

**Table 1:** Characteristics of the wave-5 analytic sample (*N* = 120,000): unweighted sample composition and survey-weighted estimates.

Characteristic	Unweighted %	Weighted %
Women	62.7	— ^a^
Age, years: mean ± SD	57.7 ± 14.4	— ^a^
Age, years: median (range)	60 (18–99)	— ^a^
Age ≤ 54	39.0	— ^a^
Higher education	48.0	— ^a^
Urban residence (≥100,000)	37.7	— ^a^
BMI ≥ 25	63.9	— ^a^
≥2 chronic conditions	64.3	— ^a^
Current smoker	20.3	— ^a^
**Affective and symptom burden**		
Lifetime physician-diagnosed depression	18.2	20.0
Poor self-rated mental health	37.0	44.5
Frequent stress	46.0	53.9
Frequent insomnia	27.2	24.8
Frequent work exhaustion	26.1	33.0
Frequent memory problems	12.2	12.2
**Supplement and OTC use**		
Any supplement use, past year	65.8	65.4
Daily use	28.6	26.9
Frequent use (FIDS)	43.2	42.3
Mood-targeted	7.2	9.6
Sleep-targeted	9.8	9.2
Memory-targeted	9.5	9.6
OTC self-medication	22.8	29.3
Management on physician’s advice	46.7	42.0
Primary-care visit <6 months	66.4	60.4

^a^ Age, sex, education, and place of residence are sample-composition/calibration variables; the weights calibrate the sample toward the margins of the adult Polish internet-using population (Mediapanel benchmarks) on these, a separate weighted estimate is therefore uninformative. Weighted estimates are shown for the health, symptom, and supplement-use variables.

**Table 2:** Survey-weighted associations between mental-health/symptom exposures and purpose-specific supplement use (primary analysis; aOR, 95% CI; each exposure in a separate model; full covariate set; *N* = 120,000). Estimates are not mutually adjusted and do not represent independent contributions. Bold = on-purpose exposure–product pairings.

Exposure	Mood Product	Sleep Product	Memory Product
Depression (lifetime diagnosis)	**2.53 (2.41–2.65)**	1.92 (1.83–2.01)	1.77 (1.69–1.85)
Poor self-rated mental health	**2.44 (2.34–2.55)**	2.04 (1.96–2.13)	1.57 (1.51–1.63)
Frequent stress	**1.80 (1.72–1.88)**	1.61 (1.54–1.68)	1.49 (1.43–1.55)
Work exhaustion	**2.17 (2.08–2.26)**	1.68 (1.61–1.75)	1.70 (1.63–1.78)
Frequent insomnia	1.40 (1.34–1.46)	**4.49 (4.31–4.69)**	1.25 (1.19–1.30)
Frequent memory problems	1.39 (1.31–1.47)	1.63 (1.54–1.71)	**2.31 (2.20–2.43)**

## Data Availability

Individual-level data cannot be shared owing to the terms of participant consent and ethics approval; aggregate results are available within the article and Supplementary Materials.

## Supplementary Materials

The following supporting information can be downloaded at https://www.mdpi.com/article/10.3390/nu18213615/s1, Table S1: Full distribution of supplement-use frequency (*N* = 120,000); Table S2: Unweighted associations between exposures and purpose-specific use (sensitivity analysis; aOR, 95% CI; *N* = 120,000); Table S3: Multivariable model for frequent supplement use (FIDS; unweighted; *N* = 120,000; aOR, 95% CI); Table S4a: Core affective associations by age stratum (unweighted; aOR, 95% CI); Table S4b: Core affective associations by sex × age cell (unweighted; aOR); Table S5: Sensitivity analyses for the principal associations.

## Abbreviations

The following abbreviations are used in this manuscript:

aOR	adjusted odds ratio
AUC	area under the curve
BMI	body mass index
CAWI	computer-assisted web interviewing
CI	confidence interval
COVID-19	coronavirus disease 2019
EPA	eicosapentaenoic acid
EZOP	Epidemiology of Mental Disorders and Access to Mental Health Care study
FIDS	frequent intake of dietary supplements
NHANES	National Health and Nutrition Examination Survey
NTZP	Narodowy Test Zdrowia Polaków (National Test of Poles’ Health)
OR	odds ratio
OTC	over-the-counter
STROBE	Strengthening the Reporting of Observational Studies in Epidemiology
