# Supplementary material for: Stress, Depression, and Purpose-Specific Use of Dietary Supplements and Over-the-Counter Preparations: A Cross-Sectional Study of 120,000 Polish Adults
Source: Nutrients. 2026 Oct 30;18(21):3615. doi: 10.3390/nu18213615 (PMC13765259; doi:10.3390/nu18213615)
Supplement: Supplementary file 1 [file nutrients-18-03615-s001.zip › nutrients-4568511-supplementary.pdf]

Supplementary Material

Stress, Depression, and Purpose-Specific Use of Dietary Supplements and Over-the-Counter Preparations: A Cross-Sectional Study of 120,000 Polish Adults

Supplementary Table S1. Full distribution of supplement-use frequency (N = 120,000).

Code	Category	n	% (unweighted / weighted)
1	Daily	34,347	28.6 / 26.9
2	Several times a week	17,536	14.6 / 15.4
3	Once a week	2,453	2.0 / 2.2
4	Several times a month	6,238	5.2 / 5.9
5	Once a month	1,369	1.1 / 1.3
6	Less than monthly	3,743	3.1 / 3.1
7	Only for a defined period	13,313	11.1 / 10.5
8	No use (past year)	41,001	34.2 / 34.7
	**Total**	**120,000**	**100 / 100**

Frequent use (FIDS) = codes 1–2 (43.2% unweighted); any use = codes 1–7 (65.8%). 0% missing.

Supplementary Table S2. Unweighted associations between exposures and purpose-specific use (sensitivity analysis; aOR, 95% CI; N = 120,000). Bold = on-purpose pairings

Exposure	Mood	Sleep	Memory
Depression (lifetime diagnosis)	**2.73 (2.60–2.88)**	1.80 (1.72–1.89)	1.65 (1.57–1.73)
Poor self-rated mental health	**2.65 (2.53–2.78)**	1.93 (1.85–2.01)	1.53 (1.47–1.59)
Frequent stress	**1.96 (1.86–2.05)**	1.52 (1.46–1.58)	1.43 (1.37–1.49)
Work exhaustion	**2.24 (2.14–2.35)**	1.65 (1.58–1.72)	1.58 (1.51–1.65)
Frequent insomnia	1.49 (1.42–1.56)	**5.02 (4.82–5.24)**	1.21 (1.15–1.26)
Frequent memory problems	1.55 (1.46–1.64)	1.60 (1.52–1.69)	**2.43 (2.32–2.56)**

Supplementary Table S3. Multivariable model for frequent supplement use (FIDS; unweighted; N = 120,000; aOR, 95% CI).

Predictor	aOR (95% CI)	Predictor	aOR (95% CI)
Female	1.45 (1.41–1.48)	Higher education	1.40 (1.36–1.43)
Age ≤ 54	1.26 (1.22–1.29)	Physical activity ≥ 1 h/week	1.39 (1.35–1.42)

Predictor	aOR (95% CI)	Predictor	aOR (95% CI)
Depression (lifetime dx)	1.19 (1.15–1.23)	≥ 2 chronic conditions	1.16 (1.13–1.19)
Poor self-rated mental health	1.10 (1.07–1.13)	Current smoking	0.71 (0.69–0.74)
Work exhaustion	1.12 (1.08–1.16)	Frequent insomnia	1.07 (1.04–1.10)
Frequent memory problems	1.09 (1.05–1.13)	BMI ≥ 25	0.86 (0.84–0.89)

Discrimination AUC = 0.64; Hosmer–Lemeshow χ^2 = 16.0, df = 8, p = 0.04 (oversensitive at this sample size; calibration practically adequate). The model is explanatory rather than predictive. Survey-weighted depression association: aOR 1.16 (95% CI 1.12–1.20).

Supplementary Table S4a. Core affective associations by age stratum (unweighted; aOR, 95% CI).

Association	≤ 54 (N = 46,786)	> 54 (N = 73,214)
Mood ~ depression	2.46 (2.29–2.63)	3.13 (2.90–3.39)
Mood ~ poor self-rated mental health	2.60 (2.43–2.77)	2.72 (2.53–2.92)
Mood ~ work exhaustion	2.16 (2.03–2.30)	2.36 (2.18–2.55)
Sleep ~ depression	1.93 (1.79–2.08)	1.74 (1.64–1.84)
Sleep ~ insomnia (validity anchor)	4.69 (4.39–5.02)	5.21 (4.94–5.50)

Supplementary Table S4b. Core affective associations by sex × age cell (unweighted; aOR).

Association	W ≤ 54 (30,863)	W > 54 (44,387)	M ≤ 54 (15,923)	M > 54 (28,827)
Mood ~ depression	2.31	3.07	2.92	3.30
Mood ~ poor mental health	2.66	2.78	2.48	2.61
Mood ~ work exhaustion	2.09	2.31	2.33	2.47
Sleep ~ depression	1.83	1.66	2.21	2.01
Sleep ~ insomnia (anchor)	4.54	5.27	5.16	5.09

Supplementary Table S5. Sensitivity analyses for the principal associations.

E-values computed on the reported odds ratio [21]: rare-outcome formula $E = OR + \sqrt{[OR(OR-1)]}$ for the low-prevalence supplement outcomes, and the risk-ratio approximation ($RR^* = \sqrt{OR}$) for the common FIDS outcome. CI column = E-value for the confidence bound nearest the null. The E-value bounds unmeasured confounding only and does not address reverse causation.

Association	aOR (unweighted → weighted)	E-value point (unweighted → weighted)	E-value CI (unweighted → weighted)
Mood ~ depression	2.73 → 2.53	4.91 → 4.49	4.63 → 4.25
Sleep ~ insomnia	5.02 → 4.49	9.52 → 8.46	9.11 → 8.08
Memory ~ memory problems	2.43 → 2.31	4.30 → 4.04	4.07 → 3.81
FIDS ~ depression	1.19 → 1.16	1.40 → 1.36	1.35 → 1.31

Multiple testing: of 135 tests in the core pool, 118 were significant at a Benjamini-Hochberg false-discovery rate of $q < 0.05$, of which 84 also met $|OR| \geq 1.2$. Continuous vs threshold age: Δ aOR ≤ 0.12. Sex-stratified sex-sensitive products: hair/skin ~ poor self-rated mental health — women 1.05 (N = 75,250), men 1.16 (N = 44,750); potency ~ depression — women 3.74 (small, atypical cell; flagged and excluded from interpretation), men 1.44. Completeness: 0% missing on analytic variables; complete-case = full N = 120,000 in every model.
